# FoundationOne CDx detected an uncovered variant of *epidermal growth factor receptor* exon 19 deletion by Oncomine Dx target test in a patient with lung adenocarcinoma

**DOI:** 10.1016/j.rmcr.2023.101893

**Published:** 2023-07-07

**Authors:** Hiroki Takahashi, Hirokazu Ogino, Hiroki Bando, Atsushi Mitsuhashi, Yuki Tsukazaki, Yohei Yabuki, Ryohiko Ozaki, Hiroto Yoneda, Seidai Sato, Masaki Hanibuchi, Yasuhiko Nishioka

**Affiliations:** a Department of Respiratory Medicine and Rheumatology, Graduate School of Biomedical Sciences, Tokushima University, 3-18-15, Kuramoto-cho, Tokushima, 770-8503, Japan; b Department of Internal Medicine, Anan Medical Center, 6-1, Kawahara, Takarada-cho, Anan-shi, Tokushima, 774-0045, Japan; c Department of Community Medicine for Respirology, Hematology and Metabolism, Graduate School of Biomedical Sciences, Tokushima University, 3-18-15 Kuramoto-cho, Tokushima, 770-8503, Japan

**Keywords:** Lung adenocarcinoma, Oncomine dx target test, Comprehensive genomic profiling, FoundationOne CDx, Driver mutations, Epidermal growth factor receptor

## Abstract

A non-smoker woman with advanced lung adenocarcinoma was referred to us. The Oncomine Dx target test (ODxTT), a next-generation sequencing (NGS)-based hot spots panel test, did not detect any driver mutations, so we treated her with chemo-immunotherapy. After second-line chemotherapy, we performed FoundationOne CDx, a NGS-based comprehensive genomic profiling (CGP) test, and identified a rare variant of *epidermal growth factor receptor* exon 19 deletion that had not been covered by ODxTT. This case highlights the importance of considering the indication of a CGP test for patients who are likely to harbor driver mutations, even when ODxTT fails to detect any.

## 1 Introduction

Lung cancer is a leading cause of cancer-related death worldwide, and the mortality rate is still increasing. Non-small-cell lung cancer (NSCLC) accounts for approximately 85% of lung cancer cases, and the most common histology is adenocarcinoma [1].

Recently, it was reported that some NSCLC patients harbor driver mutations, such as *epidermal growth factor receptor* (*EGFR*) activating mutations and *anaplastic lymphoma kinase* (*ALK*) gene rearrangement, and tyrosine kinase inhibitors (TKIs) for the corresponding gene alterations have shown a marked clinical benefit [[2], [3], [4]]. *EGFR* activating mutations, such as exon 19 in-frame deletions and exon 21 point mutation (L858R), are predominantly found in women, non-smokers, adenocarcinoma cases, and East Asian patients [5]. Furthermore, it is reported that more than half of East Asian patients with lung adenocarcinoma have some driver mutations [6]. Therefore, it is recommended to investigate these gene alterations at the diagnosis.

Next-generation sequencing (NGS) is a large-scale sequencing technology capable of identifying multiple cancer-related gene alterations simultaneously. Several NGS-based tests, such as the Oncomine Dx target test (ODxTT) and FoundationOne CDx (F1CDx), have recently been utilized in real-world clinics for NSCLC patients. ODxTT is the first NGS panel for NSCLC approved by the US Food and Drug Administration in 2017, with subsequent approval received from the Ministry of Health, Labour and Welfare of Japan in 2019. It can evaluate 46 cancer-related genes simultaneously using DNA and RNA isolated from formalin-fixed paraffin-embedded (FFPE) specimens, so it is often utilized in clinical practice [7]. However, it is also reported that the detection rate of *EGFR* mutations with ODxTT is slightly inferior to that with conventional single-gene tests, such as peptide nucleic acid-locked nucleic acid polymerase chain reaction (PNA-LNA PCR) clamp assays and cobas EGFR mutation tests [8,9].

We herein report a case of lung adenocarcinoma with a rare variant of *EGFR* exon 19 deletion (S752_I759del). It was identified by F1CDx but not ODxTT, suggesting that it is important to understand the fundamental differences between these two NGS-based tests.

## 2 Case presentation

**Fig. 1:**
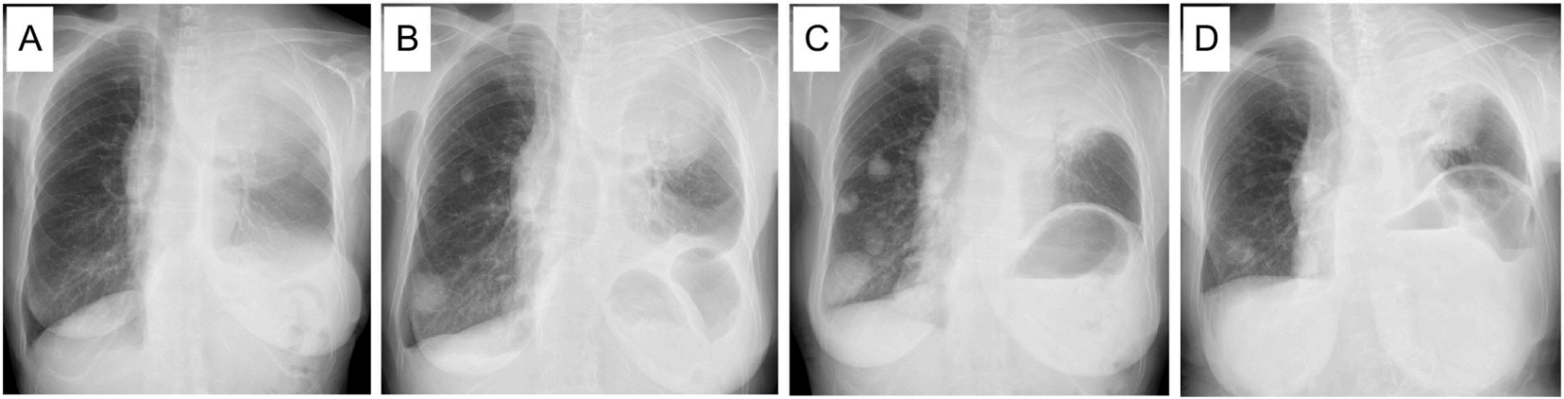
Chest X-ray findings. (A) A giant mass shadow was observed in the left upper lung field at the initial visit. (B) The pulmonary metastatic colonies in the right lung were obviously enlarged when second-line chemotherapy was introduced. F1CDx was performed at this time. (C) The lung metastatic colonies were further enlarged when osimertinib was introduced. (D) A marked anti-tumor response was observed after two months of osimertinib treatment.

**Fig. 2:**
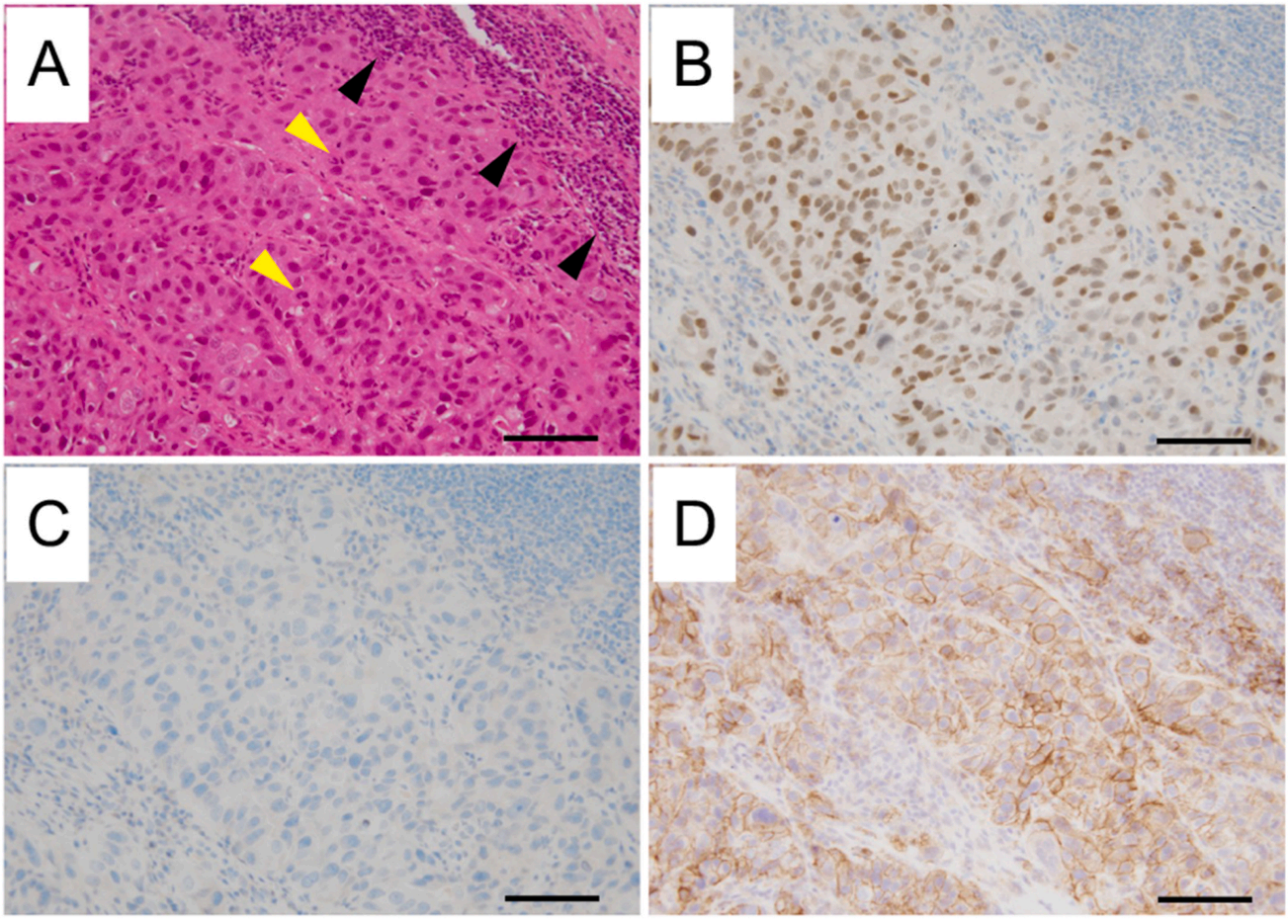
Pathological findings. (A) Hematoxylin-Eosin staining revealed that relatively enlarged malignant cells focally proliferated. Yellow arrows indicate mitotic figures. Black arrows indicate the lymphocytes infiltration. (B) The tumor cells were positive for TTF-1 (B) but negative for p40 (C). (D) PD-L1 22C3 PharmDx revealed that the tumor cells were strong positive for PD-L1 (tumor proportion score: 95%). Bars indicate 100 μm. (For interpretation of the references to colour in this figure legend, the reader is referred to the Web version of this article.)

A 74-year-old woman visited Anan Medical Center with chief complaint of severe pain in her left shoulder. X-ray showed a giant mass shadow in left upper lung field (Fig. 1A), so she was referred to Tokushima University Hospital. She had never smoked but had hypertension and dyslipidemia as concomitant diseases. Chest computed tomography (CT) revealed a huge mass shadow in her left upper lobe along with multiple pulmonary nodules and mediastinal and left supraclavicular lymph node swelling. A percutaneous biopsy of the left supraclavicular lymph node revealed that relatively enlarged malignant cells focally proliferated with severe nuclear atypia and mitotic figures in Hematoxylin-Eosin staining (Fig. 2A). The tumor cells were positive for thyroid transcription factor −1 (TTF-1) but not for p40 (Fig. 2B and C). Based on these pathological findings and imaging studies, we diagnosed her with advanced-stage lung adenocarcinoma (cT4N3M1a stage IVA) with pulmonary metastasis.

We performed the ODxTT, but it revealed that her tumor cells were negative for all driver mutations. The immunohistochemical analysis revealed that her tumor cells were positive for programmed cell death-ligand-1 (PD-L1) as determined by PD-L1 22C3 PharmDx (tumor proportion score: 95%) (Fig. 2D).

Based on these findings, we started to treat her with carboplatin (area under the curve [AUC] = 5) and pemetrexed (500 mg/m^2^) and pembrolizumab (200 mg/body); however, she developed severe erythema multiforme (Grade 3 according to the Common Terminology Criteria for Adverse Events [CTCAE] v5.0). Therefore, we were compelled to discontinue the chemotherapy and treat her with steroids. Subsequently, the primary tumor grew and invaded the spinal canal, so we treated the patient with palliative irradiation (50 Gy), followed by S-1 (50 mg twice daily for 14 consecutive days followed by 7 days of no treatment) as second-line chemotherapy, as the pulmonary metastases had obviously grown (Fig. 1B).

**Fig. 3:**
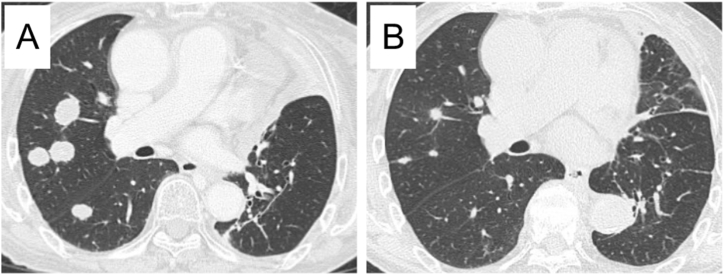
Chest computed tomography findings. Images before the administration of osimertinib showed enlarged metastatic colonies predominantly in the right lung (A). After two months of osimertinib treatment, the metastatic colonies had obviously shrunk (B).

When we started the second-line chemotherapy, we also performed F1CDx using the biopsied specimen at the diagnosis. Surprisingly, it detected a rare variant of *EGFR* exon19 deletion (S752_I759del) that was not covered by the ODxTT. Although we administered S-1 for three cycles, the metastatic nodules in her right lower lung field grew (Fig. 1C). We then treated her with osimertinib (80 mg daily), a third-generation EGFR-TKI. She showed a favorable anti-tumor response here (partial response according to the Response Evaluation Criteria in Solid Tumors [RECIST] v1.1) (Figs. 1D, and Fig. 3), and the response has continued for six months since the administration of osimertinib.

## 3 Discussion

*EGFR* mutations are well known to have a wide diversity, with more than 500 types of these mutations previously reported [10]. Although exon 19 deletions and the L858R mutation in exon 21 are dominant and account for approximately 45% and 40% of all mutations, respectively, there are also a variety of less-common mutations, termed minor mutations. Furthermore, even among exon 19 deletions, there are many variants, such as E746-A750del, L747-P753delinsS, L747-T751del, and S752_I759del [10]. As the biological activity and sensitivity to EGFR-TKIs differ among these variants, personalized treatment strategies based on the mutation subtypes are being actively explored [11].

ODxTT is categorized as a hot spot panel test and has been approved in Japan as a companion diagnostic test for targeted therapies for five driver mutations as of April 2023: *EGFR*, *ALK*, *c-ros oncogene 1* (*ROS1*), *v-raf murine sarcoma viral oncogene homolog B1* (*BRAF*), and *ret proto-oncogene* (*RET*). It can sequence the hot spots of 46 cancer-related genes based on the amplicon sequence method, which includes the amplification of each targeted site with PCR [12]. This step enables sequencing with a small amount of DNA or RNA, but it also limits the variation of detectable mutations, especially for gene rearrangements. Furthermore, the ODxTT has a fixed list of mutations and variants to be reported, meaning that it may not report the existence of some mutations that are not included in the list, even if the raw sequence data identifies them. This may be the main cause of false-negative findings by the ODxTT. Regarding *EGFR*, the current version of the ODxTT (v3.4) covers only 146 variants of *EGFR* mutations (as of February 2023), and S752_I759del, which is the variant detected in our patient, is not included in the list to be reported by the ODxTT.

Given the recent marked advances in our understanding of cancer biology, the need to evaluate genomic heterogeneity among same cancer types and driver mechanisms in different cancer types is increasing. Based on this background, comprehensive genomic profiling (CGP) approaches capable of evaluating more than 100 genes simultaneously have been developed. F1CDx is an NGS-based CGP test that can evaluate 324 cancer related genes, including mutations, amplifications, deletions, and rearrangements in the entire coding regions, along with the tumor mutation burden and microsatellite instability [13]. It utilizes the hybrid capture sequence method and reports all gene alterations, as long as their variant allele frequencies (VAFs) are ≥1% or ≥5% in the hot-spot region or entire region, respectively. This principle enables an enlarged coverage of the mutations to be reported and the detection of rare variants that cannot be detected by hot-spot panel tests, such as the ODxTT. CGP tests thus have the potential to prevent patients with driver mutations from being missed and give them the chance to receive TKIs.

In addition to these NGS-based multiplex tests, the conventional PCR-based single-gene tests (therascreen EGFR RGQ PCR assays and cobas EGFR mutation tests) and PCR-based multiplex tests (AmoyDx Pan Lung Cancer PCR panel) are also approved as companion diagnostic tests in Japan. However, it is a clinical issue that there is a disconcordance of variant coverage for EGFR mutations between each gene test. For instance, while cobas can identify S752_I759del, neither therascreen nor Amoy can identify it. Currently, CGP test can be used only for the NSCLC patients who have already received the standard chemotherapies in Japan, however it would be better to perform it for all patients to provide the optimal molecular targeted therapies without exception.

Since December 2019, when CGP tests were first approved in Japan, the number of patients receiving these tests has been rapidly increasing for several types of cancer [[14], [15], [16]]. Kunimasa et al. reported that CGP tests led to treatment with molecular-targeted agents in 6 out of 60 patients (10%) with thoracic malignancies who underwent these tests. Furthermore, four patients with *EGFR* mutations that had not been detected by PCR-based single-gene tests were included among them [17]. Tsuda et al. also reported that F1CDx identified a rare MYH9-ROS1 fusion gene that had not been detected by the ODxTT, leading to treatment with a ROS1-TKI [18].

Although the ODxTT did not detect any driver gene alterations in our patient, we still performed a CGP test, as she had factors that were associated with driver mutations, such as being a non-smoker East-Asian female with an adenocarcinoma histology. To our knowledge, this is the first report of the F1CDx identifying a rare variant of *EGFR* exon 19 deletion not covered by the ODxTT.

It is currently impossible to provide a CGP test to all NSCLC patients. Furthermore, we can provide a CGP test only once for each patient. Therefore, we consider the indication of a CGP test for the patients as follows; 1. The patients who are associated with driver mutations as described above; 2. The patients who have not previously received the multiplex gene test (regardless the histological type); 3. The patients with driver mutations who showed the acquired resistance to corresponding TKIs. However, it is desired that we can provide a CGP test to all NSCLC patients as of diagnosis or re-test for the recurrence as necessary.

In summary, we experienced a case of lung adenocarcinoma with a rare variant of *EGFR* exon 19 deletion (S752_I759del) that was detected by the F1CDx but not the ODxTT. This result led to favorable anti-tumor efficacy by treatment with osimertinib. This case suggests that the indication of a CGP test should be considered for patients who are likely to harbor driver gene alterations, even when the ODxTT does not detect any.

## Funding

This work received no specific grant from any funding agencies in the public, commercial, or not-for-profit sectors.

## Declaration of competing interest

None
